# Withdrawal and Re-treatment with Filgotinib in Ulcerative Colitis: *Post Hoc* Analyses of the Phase 2b/3 SELECTION and SELECTIONLTE Studies

**DOI:** 10.1093/ecco-jcc/jjad123

**Published:** 2023-08-04

**Authors:** Séverine Vermeire, Brian G Feagan, Laurent Peyrin-Biroulet, Alessandra Oortwijn, Margaux Faes, Angela de Haas, Gerhard Rogler

**Affiliations:** Department of Gastroenterology and Hepatology, University Hospitals Leuven, Leuven, Belgium; Alimentiv Inc., London, Ontario, Canada; Division of Gastroenterology, Department of Medicine, Western University, London, Ontario, Canada; University of Lorraine, Inserm, NGERE, Nancy, France; The Ambroise Paré-Hartmann Private Hospital Group, Paris IBD Centre, Neuilly sur Seine, France; Galapagos NV, Leiden, Netherlands; Galapagos NV, Mechelen, Belgium; Galapagos NV, Leiden, Netherlands; Department of Gastroenterology and Hepatology, University Hospital Zurich, University of Zurich, Zurich, Switzerland

**Keywords:** Filgotinib, re-treatment, ulcerative colitis

## Abstract

**Background and Aims:**

Maintenance treatment for ulcerative colitis may be discontinued for multiple reasons. This *post hoc* analysis assessed the efficacy and safety of re-treatment with filgotinib, an oral, once-daily, Janus kinase 1 preferential inhibitor, in the phase 2b/3 SELECTION trial and its long-term extension [LTE] study in ulcerative colitis.

**Methods:**

Partial Mayo Clinic Score [pMCS] response and remission were evaluated in patients who received induction with filgotinib 200 mg [FIL200] or 100 mg [FIL100], were randomized to treatment withdrawal [placebo] during maintenance, and following disease worsening, were re-treated with open-label FIL200 in the LTE study. Factors were evaluated for association with pMCS remission at LTE week 12, and safety outcomes were reported.

**Results:**

Analyses included 86 patients [FIL200: *n* = 51; FIL100: *n* = 35]. Median time to disease worsening following treatment withdrawal was 15.1 weeks (95% confidence interval [CI]: 9.1–18.7) for FIL200-induced patients and 9.6 weeks [95% CI: 6.3–12.0] for FIL100-induced patients. Three-quarters [75%] of patients achieved a pMCS response within 4–5 weeks of re-treatment in both groups. At LTE week 48, pMCS remission was achieved by 45.1% and 51.4% of FIL200- and FIL100-induced patients, respectively. Factors independently associated with restoring efficacy included no concomitant use of corticosteroids at induction baseline, and high albumin levels, pMCS remission, and endoscopic score at maintenance baseline. No new safety signals were reported among re-treated patients.

**Conclusions:**

In induction responders, re-treatment with FIL200 following temporary withdrawal from therapy restores response and/or remission in the majority of patients within 12 weeks. Re-treatment is well-tolerated. ClinicalTrials.gov identifiers: NCT02914522, NCT02914535

## Graphical Abstract

**Graphical Abstract:**
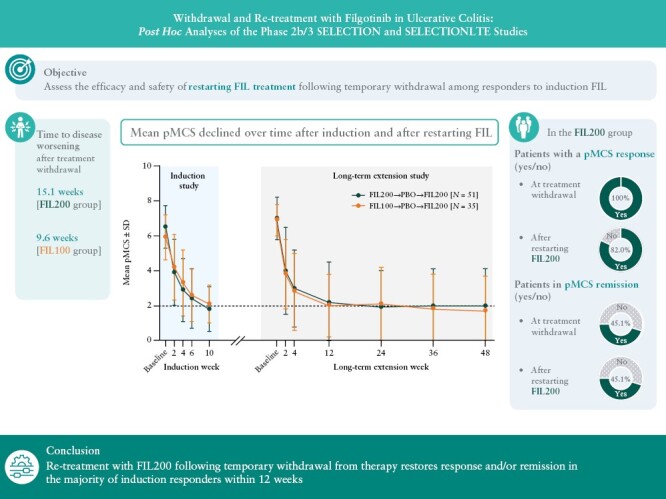

## 1. Introduction

Long-term treatment strategies are essential for the effective management of inflammatory bowel disease [IBD]. For ulcerative colitis [UC], the long-term treatment targets recommended by the recent STRIDE-II guidelines include endoscopic healing, absence of disability, and normalized health-related quality of life.^1^ Maintenance therapy helps to preserve health-related quality of life, reduces relapse rates, and potentially reduces the rates of disease-related complications, including hospitalization and surgery.^2,3^ Accordingly, strategies to enhance long-term adherence to medical maintenance regimens are needed.

There are multiple scenarios in which patients and their physicians may decide to discontinue therapy, including withdrawal due to infection, surgery, pregnancy, or comorbidities.^4^ Furthermore, the patient or physician may decide to stop treatment while the patient is in stable remission.^4^ In this case, the ‘therapeutic benefit’ of drug withdrawal relative to continued treatment is based on patient risk factors for disease complications, and the duration and quality of remission [including symptomatic, endoscopic, histological, and biomarker-based remission].^5,6^ Concerns regarding therapy withdrawal include the risk of failing to recapture remission after disease exacerbation, development of serious complications, and, in the case of biologic therapy, commensurate loss of response and risk of hypersensitivity reactions following re-treatment.^5^

Treatment options for patients with moderately to severely active UC in whom conventional therapies fail include biologics [tumour necrosis factor antagonists, anti-α4β7-integrin, anti-interleukin-12/23] and new classes of small molecules, such as Janus kinase [JAK] inhibitors and sphingosine-1 phosphate receptor modulating therapies.^7^ Filgotinib is an oral, once-daily, JAK1 preferential inhibitor indicated for the treatment of UC in the European Union, Japan, and the UK.^8,9^ The efficacy and safety of filgotinib in patients with moderately to severely active UC have been evaluated in the SELECTION study.^10^ SELECTION was a phase 2b/3 multicentre, double-blind, randomized, placebo-controlled clinical trial comprising two induction studies and a maintenance study. In SELECTION, filgotinib 200 mg was well-tolerated and efficacious in inducing and maintaining clinical remission compared with placebo in adults with moderately to severely active UC.^10^ Eligible patients who participated in SELECTION could enter the ongoing long-term extension [LTE] study, SELECTIONLTE. The *post hoc* analyses described herein assessed the efficacy and safety of re-treatment with filgotinib in patients who experienced disease worsening after temporarily discontinuing therapy.

## 2. Methods

### 2.1. Study design

Details of the study design of SELECTION [ClinicalTrials.gov ID: NCT02914522] and full eligibility criteria for enrolment have been previously described by Feagan *et al*.^10^ In SELECTION, eligible patients with moderately to severely active UC were enrolled into Induction Study A [biologic-naive patients] or Induction Study B [biologic-experienced patients]. Patients were randomized 2:2:1 to receive filgotinib 200 mg, filgotinib 100 mg or placebo orally once daily in the induction study. Patients in the filgotinib groups who were in clinical remission or had a Mayo Clinic Score [MCS] response at week 10 were re-randomized 2:1 at week 11 to continue their assigned filgotinib regimen or to receive placebo in the maintenance study until week 58 [induction placebo responders continued receiving placebo in the maintenance study]. Clinical remission was defined as a Mayo endoscopic subscore [ES] of 0 or 1, rectal bleeding [RB] subscore of 0, and at least a 1-point decrease in stool frequency [SF] from induction baseline to achieve a subscore of 0 or 1. An MCS response was defined as a reduction of at least 3 points in MCS and at least 30% from induction baseline, with an accompanying decrease in RB subscore of at least 1 point or an absolute RB subscore of 0 or 1.

Patients who completed the SELECTION study [both the induction and maintenance studies] could enter SELECTIONLTE [ClinicalTrials.gov ID: NCT02914535] to continue blinded dosing. Dosing was unblinded when the last patient completed SELECTION. Non-responders to treatment [defined as patients without clinical remission or an MCS response at week 10] received open-label filgotinib 200 mg in the LTE study. Male, non-dual refractory patients [defined as those in whom tumour necrosis factor-α antagonist and vedolizumab treatment did not fail] in the USA and the Republic of Korea were offered open-label filgotinib 100 mg in the LTE study. Patients with protocol-specified disease worsening in the SELECTION maintenance study [weeks 11–58] discontinued blinded treatment and were offered open-label filgotinib 200 mg in SELECTIONLTE. Disease worsening was defined as a partial MCS [pMCS] increase of at least 3 points to more than 5 points from the week 10 value on two consecutive visits, or an increase to 9 points on two consecutive visits if the week 10 value was more than 6. The pMCS was defined as the sum of RB, SF,and physician global assessment [PGA] subscores [i.e. all components of the MCS, except for endoscopic subscore], with a total score ranging from 0 to 9. A visit was defined as either a study visit or an unscheduled visit [any time from week 11 onwards]. Patients with disease worsening who clinically required medications prohibited by the study did not qualify for SELECTIONLTE. Prohibited medications have been previously reported by Feagan *et al*.^10^

The SELECTION and SELECTIONLTE studies were conducted in accordance with the International Conference on Harmonisation Good Clinical Practice guidelines and the Declaration of Helsinki. All patients provided written informed consent prior to inclusion.

This work was an interim analysis of the SELECTIONLTE study. Data included in these *post hoc* analyses had a cut-off date of February 24, 2022.

### 2.2. Participants

For these *post hoc* analyses, data were collected from patients treated with filgotinib 200 mg or 100 mg in the induction studies, who were randomized to receive placebo [treatment withdrawal] in the maintenance study, and, upon disease worsening, were re-treated with open-label filgotinib 200 mg in the LTE study.

### 2.3. Outcome measures and assessments

Patient clinical characteristics were evaluated at maintenance baseline. The pMCS was assessed during the induction study at baseline and weeks 2, 4, 6, and 10, and during the LTE study at baseline and weeks 2, 4, 12, 24, 36, and 48. RB and SF data were recorded by patients daily using an eDiary. The proportion of patients with a pMCS response and remission was assessed at maintenance baseline and at weeks 2, 4, 12, 24, 36, and 48 in SELECTIONLTE. A pMCS response was defined as a reduction of at least 2 points in pMCS and a reduction of at least 30% from the induction baseline [for pMCS response status at maintenance baseline] or LTE baseline [for pMCS response status in SELECTIONLTE], and pMCS remission was defined as achieving a pMCS of not more than 1. Only patients with a pMCS of at least 2 points at LTE baseline were included in the analysis for pMCS response during the LTE study. An alternative definition of pMCS remission [pMCS of not more than 2 and no RB] was used in a sensitivity analysis. Time to pMCS response and time to pMCS remission were assessed at weeks 2, 4, 12, 24, 36, 48, and 60 in SELECTIONLTE, analysed by treatment sequence.

Safety assessments included treatment-emergent adverse events [TEAEs], TEAEs related to study drug, serious TEAEs, serious TEAEs related to study drug, and TEAEs leading to temporary or permanent discontinuation of study drug. A TEAE was defined as any adverse event [AE] with an onset date on or after the study drug start date in the LTE study and no later than 30 days after permanent discontinuation of the study drug, or as any AE leading to premature discontinuation of the study drug. AE severity was graded according to the modified Common Terminology Criteria for Adverse Events, version 4.03.

### 2.4. Statistical analyses

Efficacy and safety endpoint analyses were conducted using the safety analysis set, which included patients with disease worsening who took at least one dose of the study drug in SELECTIONLTE. Descriptive statistics [*n*, %] were conducted by treatment sequence for pMCS response and pMCS remission data analyses. For time-to-event analyses, estimates of the median time to response or remission associated with each treatment group, and the 25th and 75th percentiles were obtained using Kaplan–Meier [KM] estimates. The Brookmeyer and Crowley method was used to estimate two-sided 95% confidence intervals [CIs] for median time and the interquartile range for time to event. Time to event or time to censoring was calculated from the first dosing date in the LTE study. Censored data were defined as data from patients who discontinued the study without an event before discontinuation.

For pMCS over time, a last observation carried forward imputation approach was used. For pMCS response and remission, all missing data were handled through a non-responder imputation approach. Patients who did not meet pMCS response or remission criteria owing to treatment discontinuation were considered as not achieving response or remission for that visit or any thereafter. Univariate and multivariate analyses were conducted to evaluate associations between disease characteristics and pMCS remission at week 12. Both the univariate and multivariate logistic models were fitted with the pre-specified factor, pMCS at LTE baseline, and induction dose [200 mg and 100 mg] as the covariates. For each factor in the univariate model, the odds ratio [OR] with 95% CI and overall type III *p* value were calculated. Factors with an overall type III *p* value of <0.05 were eligible for inclusion in the multivariate model. For each factor in the multivariate model, the OR with 95% CI and *p* value were calculated. All significance values are nominal because these are *post hoc* analyses.

## 3. Results

### 3.1. Patient disposition and baseline characteristics

**Figure 1.**
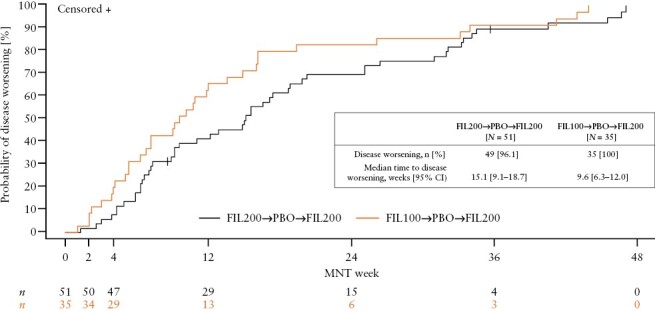
Time to disease worsening during the maintenance study. *N* is the number of patients analysed in each treatment sequence and *n* represents the number of patients at risk of the event [patients who have not discontinued the study drug and have not yet had the event at that time point]. One patient, who was considered to have treatment failure, was censored from the analysis at the last visit before treatment failure. One patient was discontinued from the maintenance study owing to disease worsening; however, the patient did not meet all criteria for protocol-specified disease worsening. The patient was censored from the analysis at the last visit. FIL100, filgotinib 100 mg; FIL200, filgotinib 200 mg; MNT, maintenance; PBO, placebo.

These *post hoc* analyses included a total of 51 patients treated with filgotinib 200 mg and 35 patients treated with filgotinib 100 mg in the induction study, who responded and were randomized to placebo in the maintenance study, and, upon disease worsening, were re-treated with filgotinib 200 mg in the LTE study [Table 1]. Baseline demographic and clinical characteristics were similar across treatment groups. The median time to disease worsening for patients induced with filgotinib 200 mg and filgotinib 100 mg was 15.1 weeks [95% CI: 9.1–18.7] and 9.6 weeks [95% CI: 6.3–12.0], respectively [Figure 1].

**Table 1.** Demographic and clinical characteristics of patients with disease worsening at maintenance baseline.

Treatment sequence [IND → MNT → LTE]	FIL200 → PBO → FIL200	FIL100 → PBO → FIL200
	*N* = 51	*N* = 35
**Age, years**		
Mean ± SD	42.6 ± 13.0	41.1 ± 15.9
Median [Q_1_–Q_3_]	42.0 [33.0–52.0]	38.0 [27.0–53.0]
**Sex, female, *n* [%]**	27 [52.9]	18 [51.4]
**Participation in IND Study A, *n* [%]**	23 [45.1]	15 [42.9]
**Concomitant therapy, *n* [%]**		
Aminosalicylates		
Yes	37 [72.5]	28 [80.0]
No	14 [27.5]	7 [20.0]
Systemically absorbed corticosteroids		
Yes	17 [33.3]	11 [31.4]
No	34 [66.7]	24 [68.6]
Immunomodulators		
Yes	10 [19.6]	7 [20.0]
No	41 [80.4]	28 [80.0]
**pMCS status at MNT baseline**		
pMCS		
Mean ± SD	1.7 ± 1.4	2.6 ± 1.8
Median [Q_1_–Q_3_]	2.0 [0.0–3.0]	2.0 [1.0–3.0]
pMCS response,^a^*n* [%]		
Yes	51 [100]	26 [74.3]
No	0	9 [25.7]
pMCS remission,^b^*n* [%]		
Yes	23 [45.1]	12 [34.3]
No	28 [54.9]	23 [65.7]

Percentages were calculated based on the number of patients in the SELECTIONLTE safety analysis set.

pMCS ranged from 0 to 9 and was defined as the sum of rectal bleeding, stool frequency, and physician global assessment subscores.

^a^pMCS response was defined as a reduction of ≥2 points and ≥30% from induction baseline.

^b^pMCS remission was defined as achieving a pMCS of ≤1.

For use of systemic corticosteroids, only records with routes of oral, intravenous, and intramuscular were included.

FIL100, filgotinib 100 mg; FIL200, filgotinib 200 mg; IND, induction; LTE, long-term extension; MNT, maintenance; PBO, placebo; pMCS, partial Mayo Clinic Score; Q, quartile; SD, standard deviation.

### 3.2. Partial MCS during SELECTION and SELECTIONLTE

**Figure 2.**
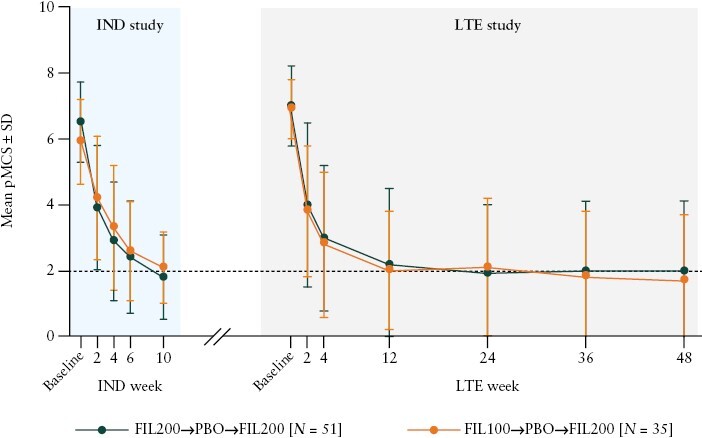
Mean pMCS measured during the induction study and the LTE study in patients induced with filgotinib 200 mg or 100 mg, withdrawn to placebo, and re-treated with filgotinib 200 mg. *N* is the number of patients analysed in each treatment sequence. A last observation carried forward imputation approach was used for the pMCS over time analysis. Data analysed at each time point were from 51 patients induced with filgotinib 200 mg and 35 patients induced with filgotinib 100 mg. The dashed line represents a pMCS value of 2, which is generally associated with good disease control. pMCS ranged from 0 to 9 and was defined as the sum of rectal bleeding, stool frequency, and physician global assessment subscores. FIL100, filgotinib 100 mg; FIL200, filgotinib 200 mg; IND, induction; LTE, long-term extension; PBO, placebo; pMCS, partial Mayo Clinic Score; SD, standard deviation.

At induction baseline, patients induced with filgotinib 200 mg and filgotinib 100 mg had a mean pMCS of 6.5 and 5.9, respectively [Figure 2]. The mean pMCS decreased rapidly in both groups from induction baseline to week 10, at which point the mean pMCS was 1.8 for the filgotinib 200 mg group and 2.1 for the filgotinib 100 mg group. At maintenance baseline, the mean pMCS was 1.7 for the filgotinib 200 mg group and 2.6 for the filgotinib 100 mg group. Following a period of therapy withdrawal, the mean pMCS of both groups at the LTE baseline exceeded those at the induction baseline [7.0 for the filgotinib 200 mg group and 6.9 for the filgotinib 100 mg group]. After 2 weeks of re-treatment with filgotinib, the mean pMCS declined rapidly in both groups [4.0 for the filgotinib 200 mg group and 3.8 for the filgotinib 100 mg group at LTE week 2]. The mean pMCS continued to decline thereafter, reaching 2.0 and 1.7 for the filgotinib 200 mg group and filgotinib 100 mg group, respectively, at week 48.

### 3.3. Partial MCS response during the LTE study

**Figure 3.**
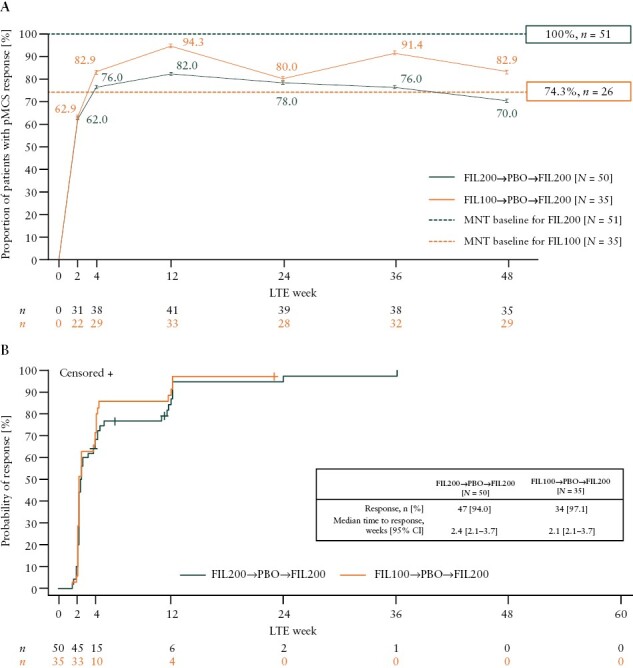
[A] Proportion of patients who achieved pMCS response over time. [B] Time to achieve pMCS response analysed through to week 60. In panel [A], *N* is the number of patients analysed in each treatment sequence and *n* is the number of patients with a pMCS response at each time point. The dashed lines represent the proportion of patients with a pMCS response at maintenance baseline. In panel [B], *n* represents the number of patients at risk of the event [patients who have not discontinued the study drug and have not yet achieved the event at that time point]. pMCS ranged from 0 to 9 and was defined as the sum of rectal bleeding, stool frequency, and physician global assessment subscores. pMCS response was defined as a reduction of ≥2 points and ≥30% from the LTE baseline. The analysis of pMCS response includes patients with pMCS of ≥2 at LTE baseline. One patient in the FIL200 group was excluded from this analysis because the patient had a pMCS of <2 at LTE baseline. FIL100, filgotinib 100 mg; FIL200, filgotinib 200 mg; LTE, long-term extension; MNT, maintenance; PBO, placebo; pMCS, partial Mayo Clinic Score.

All patients treated with filgotinib 200 mg and 74.3% of those treated with filgotinib 100 mg in the induction study achieved a pMCS response at maintenance baseline [Figure 3A]. Upon re-treatment with filgotinib 200 mg, over 60% of patients in both groups achieved a pMCS response as early as week 2 of the LTE study [62.0% for the filgotinib 200 mg group and 62.9% for the filgotinib 100 mg group]. The proportion of patients achieving a pMCS response increased at each follow-up visit up to week 12, when 82.0% of the filgotinib 200 mg group and 94.3% of the filgotinib 100 mg group achieved a pMCS response.

### 3.4. Time to pMCS response during the LTE study

The KM estimate of median time to pMCS response was 2.4 weeks [95% CI: 2.1–3.7] in patients induced with filgotinib 200 mg and 2.1 weeks [95% CI: 2.1–3.7] in those induced with filgotinib 100 mg [Figure 3B]. Three-quarters [75%; Q3] of patients achieved a pMCS response within 4.9 and 4.0 weeks of re-treatment in the filgotinib 200 mg and filgotinib 100 mg groups, respectively.

### 3.5. Partial MCS remission during the LTE study

**Figure 4.**
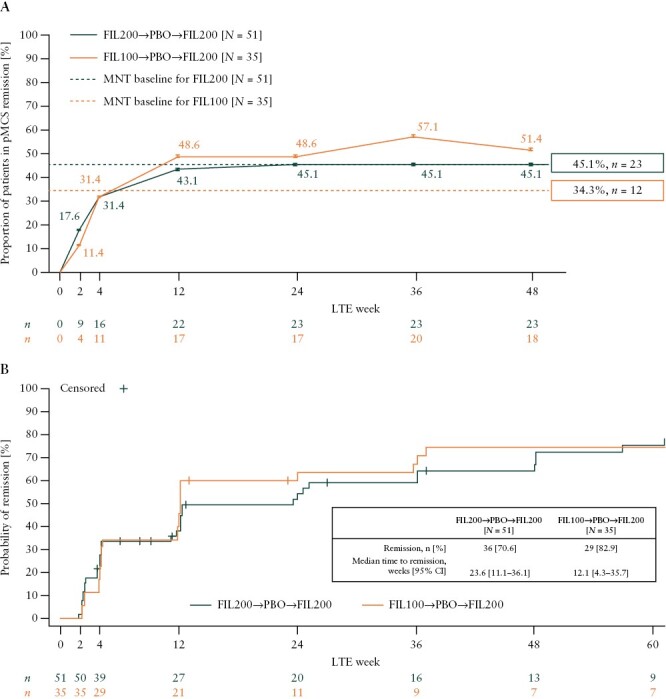
[A] Proportion of patients who achieved pMCS remission over time. [B] Time to achieve pMCS remission analysed through to week 60. In panel [A], *N* is the number of patients analysed in each treatment sequence and *n* is the number of patients in pMCS remission at each time point. The dashed lines represent the proportion of patients in pMCS remission at maintenance baseline. In panel [B], *n* represents the number of patients at risk of the event [patients who have not discontinued the study drug and have not yet achieved the event at that time point]. pMCS ranged from 0 to 9 and was defined as the sum of rectal bleeding, stool frequency, and physician global assessment subscores. pMCS remission was defined as achieving a pMCS of ≤1. FIL100, filgotinib 100 mg; FIL200, filgotinib 200 mg; LTE, long-term extension; MNT, maintenance; PBO, placebo; pMCS, partial Mayo Clinic Score.

Achievement of pMCS remission was observed in 45.1% of patients induced with filgotinib 200 mg and in 34.3% of patients induced with filgotinib 100 mg at maintenance baseline [Figure 4A]. The proportion of patients who were induced with filgotinib 200 mg in the induction study and achieved pMCS remission in the LTE study increased from 17.6% at week 2 to 45.1% at week 48, reaching the rates of remission reported for maintenance baseline. Similarly, the proportion of patients who were induced with filgotinib 100 mg in the induction study and achieved pMCS remission in the LTE study increased from 11.4% at week 2 to 51.4% at week 48. Therefore, in the filgotinib 100 mg group, the rates of pMCS remission at week 48 in the LTE study exceeded those reported for maintenance baseline.

More patients were found to have achieved pMCS remission with the alternative definition of remission than the original definition [Supplementary Figure 1A]. At maintenance baseline, 66.7% of patients induced with filgotinib 200 mg and 51.4% of patients induced with filgotinib 100 mg achieved pMCS remission. From week 2 to week 48, there was an increase in the proportion of patients achieving pMCS remission in both groups [from 35.3 to 56.9% in the filgotinib 200 mg group and from 28.6 to 65.7% in the filgotinib 100 mg group].

### 3.6. Time to pMCS remission during the LTE study

The KM estimate of median time to pMCS remission was 23.6 weeks [95% CI: 11.1–36.1] and 12.1 weeks [95% CI: 4.3–35.7] in the filgotinib 200 mg and filgotinib 100 mg groups, respectively [Figure 4B]. Within the period analysed, 70.6% of patients induced with filgotinib 200 mg achieved pMCS remission in at least one visit and 29.4% of this patient group did not achieve pMCS remission. Of the patients induced with filgotinib 100 mg, 82.9% achieved pMCS remission in at least one visit within the period analysed, and 17.1% did not.

The median time to pMCS remission using the alternative definition was estimated to be 4.4 weeks [95% CI: 2.6–12.1] and 11.7 weeks [95% CI: 3.9–12.1] in the filgotinib 200 mg and filgotinib 100 mg groups, respectively [Supplementary Figure 1B]. Within the period analysed, 82.4% of patients induced with filgotinib 200 mg had achieved pMCS remission in at least one visit and 17.6% of this patient group had not achieved pMCS remission. Of the patients induced with filgotinib 100 mg, 88.6% had achieved pMCS remission in at least one visit within the period analysed, and 11.4% had not.

### 3.7. Analysis of predictors of achievement of pMCS remission upon re-treatment

**Figure 5.**
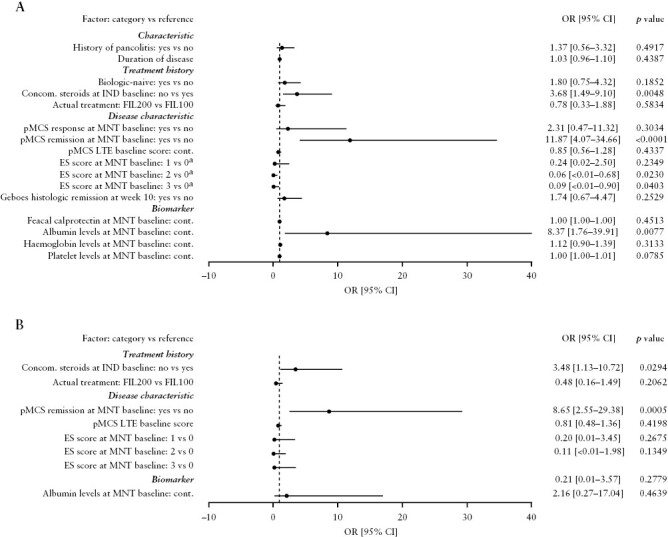
Factors assessed using [A] univariate and [B] multivariate analyses for their association with pMCS remission at week 12 of re-treatment. ^a^The overall type III *p* value is equal to 0.0378. CI, confidence interval; ES, endoscopic subscore; FIL100, filgotinib 100 mg; FIL200, filgotinib 200 mg; IND, induction; LTE, long-term extension; MNT, maintenance; OR, odds ratio; pMCS, partial Mayo Clinic Score.

In the univariate model, lack of concomitant use of corticosteroids at induction baseline [OR: 3.68; 95% CI: 1.49–9.10; *p *= 0.0048] was positively associated with pMCS remission at week 12 of re-treatment [Figure 5A]. In addition, pMCS remission [OR: 11.87; 95% CI: 4.07–34.66; *p *< 0.0001] at maintenance baseline was positively associated with pMCS remission at week 12 of re-treatment. An endoscopic subscore of at least 1 [overall type III *p *= 0.0378] at maintenance baseline was negatively associated with pMCS remission at week 12. High albumin levels [OR: 8.37; 95% CI: 1.76–39.91; *p *= 0.0077] were positively associated with pMCS remission at week 12, whereas high faecal calprotectin levels and high platelet levels were not. In the multivariate model, lack of concomitant use of corticosteroids at induction baseline [OR: 3.48; 95% CI: 1.13–10.72; *p *= 0.0294] and pMCS remission at maintenance baseline [OR: 8.65; 95% CI: 2.55–29.38; *p *= 0.0005] were positively associated with pMCS remission at week 12 of re-treatment [Figure 5B].

### 3.8. Safety endpoints

TEAEs were experienced by 78.4% of patients induced with filgotinib 200 mg and 80.0% of patients induced with filgotinib 100 mg [Table 2]. TEAEs related to filgotinib were reported in 15.7% of patients in the filgotinib 200 mg group and 25.7% of patients in the filgotinib 100 mg group. Few patients in either group experienced serious TEAEs (nine patients [17.6%] in the filgotinib 200 mg group and four patients [11.4%] in the filgotinib 100 mg group). In the filgotinib 200 mg group, the serious TEAEs reported were diabetes mellitus [*n* = 1], COVID-19 [*n* = 2], COVID-19 pneumonia [*n* = 1], infective bursitis [*n* = 1], Meniere’s disease [*n* = 1], osteoarthritis [*n* = 1], sacroiliitis [*n* = 1], and UC [*n* = 3]. In the filgotinib 100 mg group, the serious TEAEs reported were adrenal insufficiency [*n* = 1], adrenal neoplasm [*n* = 2], cholelithiasis [*n* = 1], hypertension [*n* = 1], pelvic cyst [*n* = 1], and pneumonia [*n* = 1]. Overall, two serious TEAEs [adrenal insufficiency, adrenal neoplasm] were not resolved within the period analysed; both occurred in the filgotinib 100 mg group. Serious TEAEs considered related to filgotinib by the investigator were reported in two patients [3.9%] in the filgotinib 200 mg group [COVID-19, infective bursitis] and one patient [2.9%] in the filgotinib 100 mg group [pneumonia]. Four patients [7.8%] in the filgotinib 200 mg group and eight patients [22.9%] in the filgotinib 100 mg group had TEAEs leading to temporary study drug discontinuation. In total, ten patients [19.6%] in the filgotinib 200 mg group and two patients [5.7%] in the filgotinib 100 mg group had TEAEs leading to premature study drug discontinuation. No TEAEs leading to death were reported. The number of AEs upon re-treatment in the LTE study was generally consistent with that reported for the SELECTION study.^10^

**Table 2.** Safety outcomes in patients re-treated with filgotinib 200 mg in the SELECTIONLTE study.

Treatment sequence [IND → MNT → LTE]	FIL200 → PBO → FIL200	FIL100 → PBO → FIL200
	*N* = 51	*N* = 35
TEAE, *n* [%]	40 [78.4]	28 [80.0]
Grade 2 or higher	31 [60.8]	20 [57.1]
Grade 3 or higher	10 [19.6]	5 [14.3]
TEAE related to study drug, *n* [%]	8 [15.7]	9 [25.7]
Serious TEAE, *n* [%]^a^	9 [17.6]	4 [11.4]
Serious TEAE related to study drug, *n* [%]	2 [3.9]	1 [2.9]
TEAE leading to temporary interruption of study drug, *n* [%]	4 [7.8]	8 [22.9]
TEAE leading to premature discontinuation of study drug, *n* [%]	10 [19.6]	2 [5.7]
Serious TEAE leading to death, *n* [%]	0 [0]	0 [0]

A TEAE was defined as any AE with an onset date on or after the study drug start date in the LTE study and no later than 30 days after permanent discontinuation of the study drug, or as any AE leading to premature discontinuation of the study drug.

^a^In the filgotinib 200 mg group, the serious TEAEs reported were diabetes mellitus [*n* = 1], COVID-19 [*n =* 2], COVID-19 pneumonia [*n =* 1], infective bursitis [*n =* 1], Meniere’s disease [*n =* 1], osteoarthritis [*n =* 1], sacroiliitis [*n =* 1], and UC [*n =* 3]. In the filgotinib 100 mg group, the serious TEAEs reported were adrenal insufficiency [*n =* 1], adrenal neoplasm [*n =* 2], cholelithiasis [*n =* 1], hypertension [*n =* 1], pelvic cyst [*n =* 1], and pneumonia [*n =* 1].

AE, adverse event; FIL100, filgotinib 100 mg; FIL200, filgotinib 200 mg; IND, induction; LTE, long-term extension; MNT, maintenance; PBO, placebo; TEAE, treatment-emergent adverse event.

## 4. Discussion

Withdrawal and re-initiation of treatment might be necessary for some patients with UC during their lifetime. This study is the first to assess withdrawal and re-treatment with filgotinib in a clinical setting; data from the SELECTION and SELECTIONLTE studies were used for the analyses. Specifically, the efficacy and safety of filgotinib 200 mg were assessed in induction [filgotinib 200 mg or filgotinib 100 mg] responders who experienced disease worsening following a period of treatment withdrawal [up to 47 weeks], and subsequently re-initiated treatment with filgotinib 200 mg. Following re-treatment, a pMCS response was reinstated as early as week 2 of re-treatment in 62.0–62.9% of patients, regardless of their induction dose. Within 12 weeks of re-treatment, more than 80% of patients regained a pMCS response. The proportions of patients achieving a pMCS response and remission generally increased throughout the 48-week period analysed. Following 48 weeks of re-treatment, the proportions of patients in pMCS remission reached or exceeded the proportions at maintenance baseline. Re-treatment with filgotinib was well tolerated.

Withdrawing maintenance treatment may be necessary to accommodate a variety of scenarios, including infection, surgery, pregnancy, or comorbidities.^4^ Moreover, clinicians occasionally consider permanent withdrawal of treatment for patients who are in stable remission to avoid adverse effects and risks that may be associated with treatment.^4^ However, high relapse rates have been reported after therapy withdrawal; for example, in prospective analyses of patients with IBD [e.g. the STORI cohort of patients with Crohn’s disease] and in clinical trials of patients with UC [e.g. OCTAVE SUSTAIN].^11–13^ In the 7-year follow-up study of the STORI cohort, only 21.6% of patients who were in remission following treatment with infliximab and immunomodulators did not restart biologic treatment and did not have a major complication following infliximab withdrawal.^14^ In our study, the median time to disease worsening upon treatment withdrawal was ~3.5 months [15.1 weeks] and 2.2 months [9.6 weeks] for patients induced with filgotinib 200 mg and filgotinib 100 mg, respectively, highlighting the importance of maintaining therapy. Together with previously published data, our findings support the need for continued treatment instead of intermittent therapy, when possible.^12^

This study showed that following treatment withdrawal, re-treatment with filgotinib 200 mg was efficacious in most [82.0–94.3%] patients following 12 weeks of re-treatment. The onset of pMCS response occurred as early as week 2 of re-treatment for both groups, and good disease control was then maintained over the course of 48 weeks in the LTE study. Within 4–5 weeks of re-treatment, 75% of patients achieved a pMCS response in both groups, indicating fast recovery of response. Following 48 weeks of re-treatment, the number of patients in pMCS remission was equal to [filgotinib 200 mg group] or exceeded [filgotinib 100 mg group] the number of patients in pMCS remission at maintenance baseline. Panes *et al*.^12^ reported that efficacy was achieved as early as 1 month after re-treatment with JAK inhibitors in patients with UC. Our findings suggest that efficacy may be restored even earlier than previously reported; however, careful consideration of study design and patient populations is needed when comparing efficacy among different studies of JAK inhibitors.

In our study, a higher rate of recapturing remission at week 12 of re-treatment was reported for patients who had either achieved pMCS remission or endoscopic remission,^10^ or who had a good nutritional status [as indicated by serum albumin levels]^15^ at the time of treatment withdrawal, compared with other patients. Patients who were previously dependent on corticosteroids had a lower rate of regaining response compared with other patients, indicating that treatment withdrawal should be avoided in this patient subgroup, if possible.

Re-treatment with filgotinib 200 mg was well tolerated, with few TEAEs leading to temporary or premature discontinuation of filgotinib. There were no new safety signals reported among re-treated patients, which is consistent with findings from the SELECTION study.^10^ Therefore, re-treatment with filgotinib may provide a suitable option for physicians and their patients when considering temporary discontinuation of therapy without the need to introduce a treatment with a different mode of action.

Patients in the filgotinib 100 mg group were re-induced with filgotinib 200 mg in SELECTIONLTE, which seemed to lead to a better initial pMCS response than those who did not change dose [filgotinib 200 mg group]; however, this is probably an artefact of dose escalation during re-treatment. Interpretation of this response is limited by the low numbers of patients in the re-treated subpopulation.

A further limitation to the low number of patients analysed herein is that these patients received open-label treatment during the SELECTIONLTE study. Furthermore, the SELECTIONLTE study is ongoing; this was an interim analysis of the LTE data. Future work could evaluate the efficacy and safety of re-treatment with filgotinib 200 mg during a longer follow-up period than in the current analysis.

In conclusion, rates of pMCS response and remission with filgotinib 200 mg after initial treatment were similar to those after re-treatment following a withdrawal period. Furthermore, re-treatment with filgotinib 200 mg was well tolerated. Collectively, these *post hoc* analyses indicate that re-treatment with filgotinib 200 mg is an effective and viable therapy option for patients who need to temporarily discontinue UC treatment.

## Supplementary Material

jjad123_suppl_Supplementary_Data

## Data Availability

Anonymized individual patient data will be shared upon request for research purposes dependent upon the nature of the request, the merit of the proposed research, the availability of the data, and their intended use. The full data sharing policy for Gilead Sciences, Inc., and Galapagos NV can be found at https://www.gilead.com/about/ethics-and-code-of-conduct/policies and https://www.clinicaltrials-glpg.com/us/en/data-transparency.html, respectively.

## Conference Presentation

ECCO 2022 [virtual], DDW 2022 [San Diego, CA, and virtual].
