# Supplementary material for: Withdrawal and Re-treatment with Filgotinib in Ulcerative Colitis: Post Hoc Analyses of the Phase 2b/3 SELECTION and SELECTIONLTE Studies
Source: J Crohns Colitis. 2023 Aug 4;18(1):54–64. doi: 10.1093/ecco-jcc/jjad123 (PMC10821704; doi:10.1093/ecco-jcc/jjad123)
Supplement: jjad123_suppl_Supplementary_Data [file jjad123_suppl_supplementary_data.docx]

# Supplementary data

## **Plain language summary (321 words)**

Ulcerative colitis [UC] is a chronic condition where part of the large intestine or bowel becomes inflamed and can lead to life-threatening complications. Common symptoms of UC include abdominal pain, frequent diarrhoea, urgently needing to empty the bowel and rectal bleeding. People with UC sometimes stop taking their medication for a variety of reasons. It is important to understand if the same medication can benefit people when they start taking it again after having stopped. Filgotinib is an oral pill that reduces inflammation to improve the symptoms of UC. In the SELECTION study, we compared filgotinib with placebo in adults with moderately to severely active UC over 58 weeks.

The results here are from 86 patients who first took filgotinib and responded to treatment, and then switched to placebo in the main part of the study. These patients then started taking filgotinib again during the long-term extension [LTE] of the study because their symptoms got worse while taking placebo. During the main study and the LTE, we calculated a score for each patient called the partial Mayo Clinic Score. The lower the score, the less severe the UC symptoms are. We determined how many patients achieved a good result based on a low score. Overall, 82.0–94.3% of these patients achieved a good score after taking filgotinib again during the LTE for 12 weeks. The patients responded quickly to taking filgotinib again and maintained their response during the LTE. We also determined how many patients had disease remission. Remission means the absence of the signs and symptoms of disease. Similar numbers of patients who were in remission when they first took filgotinib during the main part also achieved remission when they started taking filgotinib again during the LTE for 48 weeks.

Our results show that filgotinib was well-tolerated in patients who started taking it again, and that taking filgotinib again may be a suitable option for patients who initially responded to treatment but have stopped taking the drug.

## **Supplementary Figure 1.** Sensitivity analysis using the alternative definition of pMCS remission. [A] Proportion of patients who achieved pMCS remission over time. [B] Time to achieve pMCS remission analysed through to week 60.

A


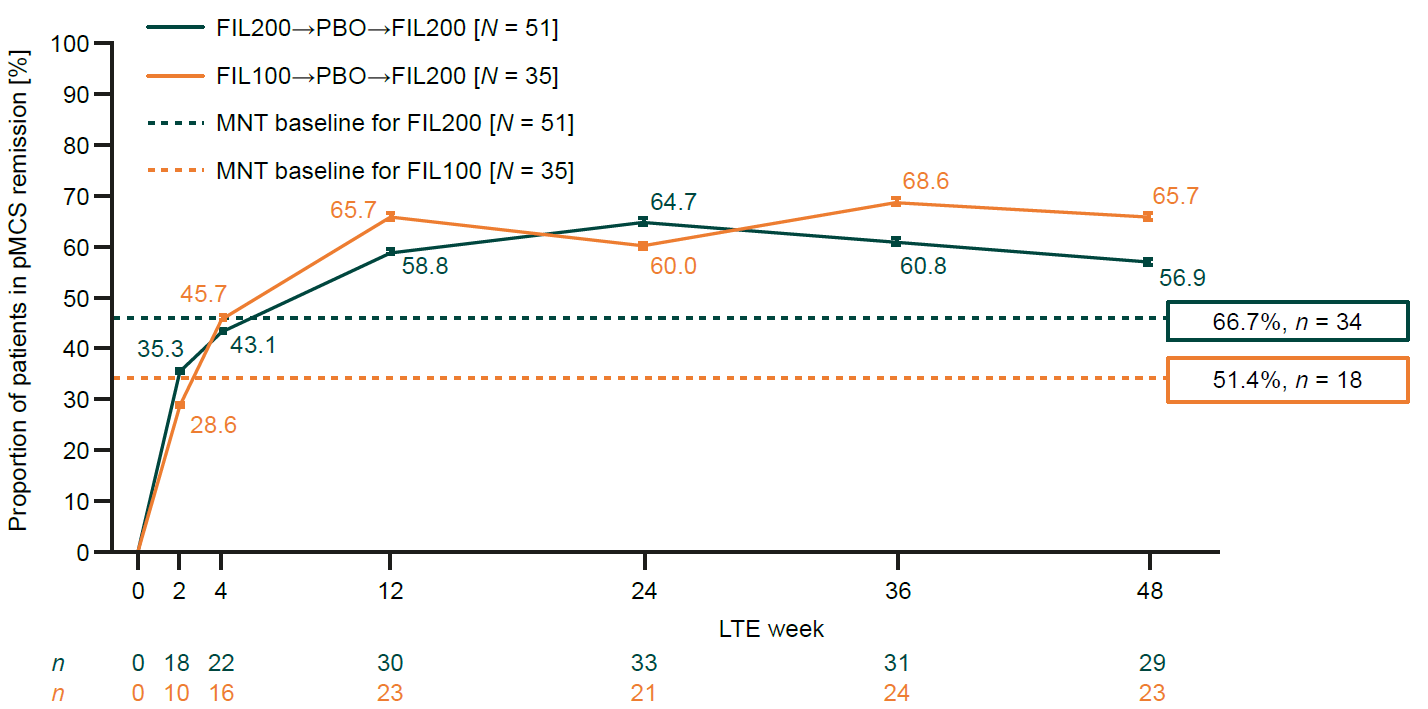


B


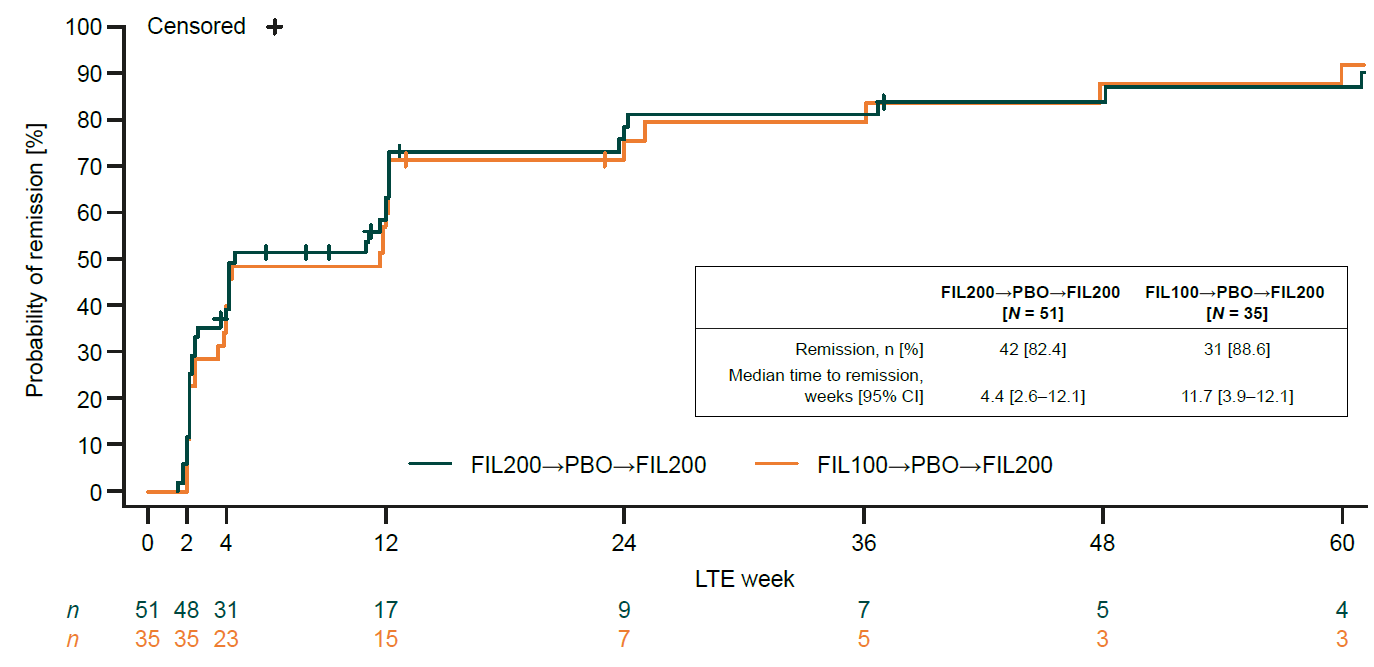


In panel [A], *N* is the number of patients analysed in each treatment sequence and *n* is the number of patients in pMCS remission at each time point. The dashed lines represent the proportion of patients in pMCS remission at maintenance baseline.
In panel [B], n represents the number of patients at risk of the event [patients who have not discontinued the study drug and have not yet achieved the event at that time point].

pMCS ranged from 0 to 9 and was defined as the sum of rectal bleeding, stool frequency and physician global assessment subscores.

pMCS remission was defined as a pMCS of ≤ 2 and no rectal bleeding.

FIL100, filgotinib 100 mg; FIL200, filgotinib 200 mg; LTE, long-term extension; MNT, maintenance; PBO, placebo; pMCS, partial Mayo Clinic Score.
